# Real-World Imaging Data: Opportunities and Challenges

**DOI:** 10.2196/88202

**Published:** 2026-07-20

**Authors:** Jie Wu, Aline L de Araujo, Sean Khozin, Merel Huisman, Domenico Mastrodicasa, Martin J Willemink

**Affiliations:** 1 Segmed, Inc, 3790 El Camino Real #810, Palo Alto, CA, 94306, United States; 2 Universidade Federal de São Paulo, São Paulo, Brazil; 3 Project Data Sphere, Cary, NC, United States; 4 CEO Roundtable on Cancer, Cary, NC, United States; 5 Department of Radiology and Nuclear Medicine, Radboud University Medical Center, Nijmegen, Gelderland, The Netherlands; 6 Department of Radiology, School of Medicine, University of Washington, Seattle, WA, United States

**Keywords:** real-world data, real-world evidence, imaging, data bias, data diversity

## Abstract

The amount of data generated in clinical practice is increasing substantially. This has benefited the use of real-world data for real-world evidence in biomedical research. While early real-world evidence efforts focused on structured electronic health record and insurance claims data, advances in analytical methods, such as AI, are expected to further enhance the ability to obtain deeper insights from real-world data. Most recently, real-world imaging data (RWiD) has emerged as a novel and valuable resource. RWiD is enabled by improvements in imaging infrastructure, data standardization, and deidentification technologies. Medical imaging is essential at multiple stages of clinical care, ranging from screening to posttreatment assessment and surveillance. Medical imaging has become a key component of patient management, as it augments clinical decision-making across many medical specialties. RWiD is the retrospective collection of routinely gathered clinical imaging data. Using only radiology reports results in limited information compared to datasets that contain the actual images. Radiology reports primarily focus on clinical decision-making rather than research purposes. Therefore, the actual images add value to real-world datasets. However, using RWiD is challenging due to complex deidentification and harmonization, as well as requirements for file storage, file transfer, and computation. This editorial paper provides an educational overview, including the background, challenges, and opportunities of RWiD, and offers examples of RWiD applications that benefit life sciences and biopharmaceutical use cases.

## Introduction

The amount of data generated in clinical practice is increasing substantially due to advances in computing, health care information technology, and policy [1,2]. Health records were historically paper-based. The Health Information Technology for Economic and Clinical Health Act, enacted into law in 2009, spurred the widespread adoption of electronic health records (EHRs) among health care providers in the United States [3,4]. The increasing availability of point-of-care clinical data in EHRs has benefited the use of real-world data (RWD) for real-world evidence (RWE) in biomedical research. RWD is observational data routinely collected in clinical settings, including EHR, health insurance claims, and patient-reported outcomes. RWD also includes data collected outside the traditional clinical setting, such as wearables and emerging data sources such as social media [5]. RWE is clinical evidence derived from RWD for a variety of applications, particularly regarding the use, effectiveness, and safety of medical products [6]. Early RWE efforts focused on structured EHR and insurance claims data [2]. Advances in analytical methods, such as AI, are expected to further enhance the ability to obtain deeper insights from RWD [7]. In this current paper, EHR data refer to structured and semistructured clinical data, including coded variables (eg, diagnoses, medications, and laboratory values) and unstructured clinical text. In parallel, more complex data types such as genomics and structured laboratory test results are increasingly integrated into RWE frameworks [8,9]. Most recently, real-world imaging data (RWiD) has emerged as a novel and valuable resource. RWiD is enabled by improvements in imaging infrastructure, data standardization, and deidentification technologies [10]. Although imaging studies are technically part of the EHR, they are treated here as a distinct data modality (RWiD) due to their unique technical, analytical, and infrastructural characteristics [11].

**Figure 1.**
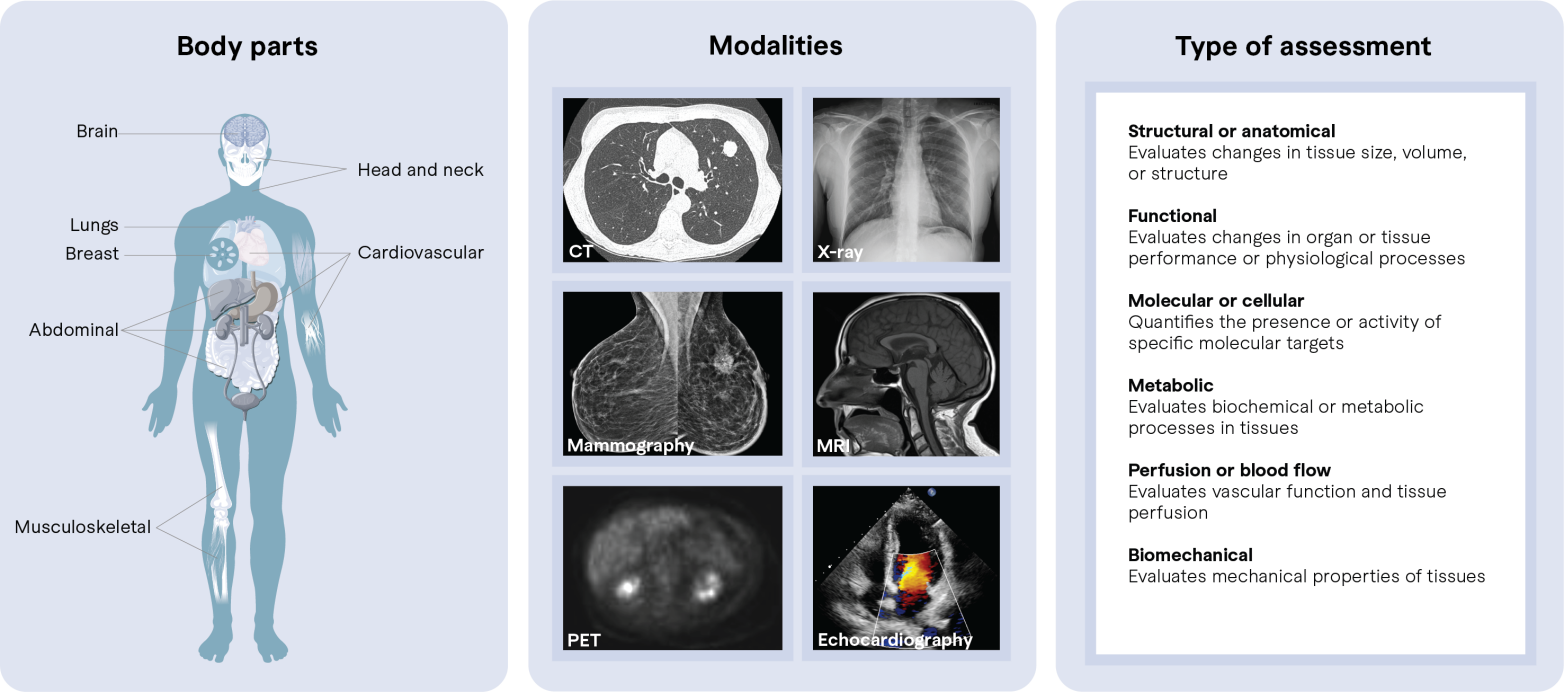
Body parts, modalities, and types of assessment for radiology images. CT: computed tomography; MRI: magnetic resonance imaging; PET: positron emission tomography.

Medical imaging plays a key role in patient management by providing more definitive diagnostic information and guiding treatment decisions across multiple medical specialties (Figure 1) [12-14]. While its applications are wide-ranging, imaging is especially critical in therapeutic areas where it plays a central role in both routine care and research, most notably in oncology, cardiology, and neurology (Table 1) [15]. Typically, medical imaging includes x-ray, computed tomography (CT), magnetic resonance imaging (MRI), and ultrasound, as well as (hybrid) nuclear medicine modalities such as positron emission tomography and single-photon emission computed tomography [16].

**Table 1.** Type of assessments enabled by imaging modalities and examples of their respective applications in clinical care.

Type of assessment, what it evaluates, and clinical domain	Examples
Structural or anatomical	
Changes in tissue size, volume, or structure	
Oncology	Tumor size/volume: RECIST^a^ criteria with CT^b^ or MRI^c^
Neurology	Brain atrophy: hippocampal volume in Alzheimer (MRI)
Cardiology	Vessel morphology: coronary artery stenosis (CT angiography)
Orthopedics	Cartilage thickness: osteoarthritis (MRI); bone microarchitecture, osteoporosis (CT)
Functional	
Changes in organ or tissue performance or physiology	
Cardiology	Cardiac function: LVEF^d^ on echocardiography or cardiac MRI
Neurology	Cerebral perfusion: CT perfusion in stroke
Respiratory	Pulmonary function: V/Q^e^ mismatch (nuclear medicine)
Molecular or cellular	
Quantify the presence or activity of specific molecular targets	
Oncology	Hypoxia markers, hypoxia imaging: PET^f^ with tracers such as FMISO^g^; HER2^h^ expression, HER2-PET in breast cancer for patient stratification
Neurology	Amyloid/tau deposition; amyloid PET in Alzheimer disease.
Metabolic	
Biochemical or metabolic processes in tissues	
Oncology	Lactate production; tumor metabolism (MR^i^ spectroscopy)
Neurology	Glucose metabolism: FDG^j^-PET in Alzheimer disease and epilepsy
Endocrinology	Fat deposition; hepatic fat fraction in nonalcoholic fatty liver disease (NAFLD^k^; MRI)
Perfusion or blood flow	
Vascular function and tissue perfusion	
Neurology	Tumor angiogenesis (DCE^l^-MRI)
Oncology	Cerebral blood flow: arterial spin labeling MRI in stroke or dementia
Biomechanical	
Mechanical properties of tissues	
Hepatology	Tissue stiffness: liver fibrosis (MRI elastography)
Cardiology	Cardiac strain: speckle-tracking echocardiography

a RECIST: response evaluation criteria in solid tumors.

b CT: computed tomography.

c MRI: magnetic resonance imaging.

d LVEF: left ventricular ejection fraction.

e V/Q: ventilation/perfusion.

f PET: positron emission tomography.

g FMISO: fluoromisonidazole.

h HER2: human epidermal growth factor receptor 2.

i MR: magnetic resonance.

j FDG: 18F-labeled fluoro-2-deoxyglucose.

k NAFLD: nonalcoholic fatty liver disease.

l DCE: dynamic contrast-enhanced.

As imaging technologies continue to evolve, the importance of imaging data in health care, both in routine clinical care and in research, is expected to grow even further. Medical imaging is foundational to clinical trials, and the emergence of RWiD is creating new opportunities for biopharmaceutical and life sciences research [17]. Medical imaging plays a central role in therapeutic development and clinical trials. In oncology, for example, standardized imaging criteria such as response evaluation criteria in solid tumors (RECIST) are widely used to evaluate treatment response and define trial endpoints [18]. More broadly, imaging provides quantitative and spatially resolved assessments of disease burden and progression. This information is difficult to capture using structured EHR or insurance claims data alone. These traditional data sources primarily reflect health care usage, diagnoses, and procedures, but often lack detailed phenotypic information, longitudinal disease characterization, and objective measures of treatment response [7]. However, while biopharmaceutical and life sciences researchers typically use traditional data modalities such as insurance claims and clinical notes (EHR), the use of RWiD is currently limited. The most important reason for the limited use is the lack of awareness and novelty of the availability of this data modality.

A recent review by Navarro-Garcia et al [19] highlights the growing role of RWiD in AI-driven biomarker development in oncology. The authors emphasize the value of large, heterogeneous imaging datasets derived from routine clinical care, while also noting key challenges related to data quality, standardization, and validation. The present editorial paper takes a broader perspective on RWiD, extending beyond AI-centric applications to include its role across the life cycle of biopharmaceutical research. In addition to biomarker discovery, we focus on practical considerations such as deidentification, data governance, and infrastructure, and outline applications in clinical trial design, external control arms, postmarketing surveillance, and drug repurposing. The goal of this editorial paper is to increase the awareness of RWiD for biopharmaceutical researchers.

### Challenges of RWD

Clinical trial data are typically structured and protocol-driven. In contrast, RWD often consists of large volumes of unstructured information [20]. Much of this data resides in fragmented, secure silos within health care systems, making access, storage, and analysis challenging [10,21]. For all its flaws in structure and standardization, EHR and insurance claims data offer practical advantages, being relatively lightweight and easier to deidentify [22,23]. However, these sources often miss critical information needed for deeper clinical insights. As a result, complex data types such as genomics and other high-dimensional modalities are leveraged to enhance analytical and evidentiary value.

### Imaging Specific Challenges

While RWiD provides valuable information in addition to other data types such as EHR and insurance claims, RWiD comes with its own challenges.

## Bias

### Challenge

Medical imaging data consists of high-resolution (eg, mammography) and/or 3D data (eg, CT and MRI) stored as digital imaging and communications in medicine (DICOM) files [24]. DICOM files consist of pixels (the actual images) and DICOM tags (metadata that provides information about the image and the equipment, among other acquisition and reconstruction settings). DICOM files need to be harmonized to reduce biases introduced by vendor, scanner type, and acquisition and reconstruction protocol differences. Vendor-specific DICOM tags also result in heterogeneity. Although DICOM provides a standardized file format for storing and exchanging imaging data, substantial variability persists in practice. Differences in scanner hardware, acquisition parameters (eg, tube voltage, slice thickness, and contrast timing), and reconstruction techniques (eg, kernel selection and iterative reconstruction algorithms) can lead to systematic differences in image appearance and quantitative measurements across sites and vendors [25,26]. In addition, vendor-specific implementations of DICOM tags and private metadata fields introduce further heterogeneity. As a result, harmonization remains necessary to improve comparability and reproducibility in multicenter imaging studies. Data harmonization tools such as ComBat can help reduce biases caused by scanner and protocol differences [27]. However, ComBat should be applied with caution (Table 2). Despite this, RWiD is inherently subject to selection bias [28]. Imaging is not performed randomly but rather driven by clinical indication, physician preference, access to care, and institutional protocols. As a result, the population captured in imaging datasets may not be representative of the broader patient population. For example, patients undergoing advanced imaging (eg, CT or MRI) often differ systematically from those who do not, in terms of disease severity, comorbidities, and socioeconomic factors. Additionally, RWD derived from tertiary or academic centers may overrepresent complex cases, further limiting the generalizability of research conducted on both RWD and RWiD.

**Table 2.** ComBat is used to mitigate scanner and protocol-related batch effects, but it should be applied with caution [29].

When not to use ComBat:	Description of when not to use ComBat
Confounded study design	Avoid ComBat if the biological variable of interest (eg, disease state) is confounded with the site/scanner. Harmonization in this context may inadvertently remove the clinical signal that is aimed to be detected.
Small sample sizes	ComBat relies on estimating the distribution of batch effects. Small cohorts can lead to unstable parameter estimates.
Out-of-distribution populations	If a site represents a unique subpopulation not captured elsewhere, global normalization may strip away meaningful biological diversity.

### Mitigation

Several strategies can help mitigate bias in RWiD. First, careful cohort design and transparent reporting of inclusion criteria are essential to assess representativeness and generalizability. Linking imaging data with complementary data sources, such as EHR and insurance claims, can provide additional clinical context and enable adjustment for measured confounders. Statistical techniques, including propensity score methods, inverse probability weighting, and stratification, may further reduce the impact of selection bias, although they cannot fully address unmeasured confounding. At the data level, harmonization approaches and standardized imaging protocols can help reduce technical variability across sites, but should be applied cautiously to avoid removing clinically meaningful variation. Finally, external validation across diverse institutions and patient populations is critical to ensure robustness and generalizability of findings derived from RWiD.

## Heterogeneity in Imaging Acquisition and Reconstruction Protocols

### Challenge

A challenge unique to RWiD is the heterogeneity in acquisition and reconstruction settings across and within institutions, vendors, and time [25]. Variability in scanner models, imaging protocols, contrast administration, and postprocessing methods can introduce systematic differences in image appearance. This variability may reduce the reproducibility and generalizability of research conducted with RWiD.

### Mitigation

Standardization of imaging protocols across sites can reduce variability, particularly in prospective studies and multicenter collaborations. When standardization is not feasible, detailed capture and use of acquisition and reconstruction metadata are essential to enable post hoc harmonization and sensitivity analyses. Statistical harmonization techniques, such as ComBat, may reduce site-specific effects but should be applied cautiously to avoid removing clinically meaningful variation. In addition, model development strategies that explicitly account for domain shift, including domain adaptation and training on multicenter datasets, can improve robustness to heterogeneity.

## File Format and Data Standardization

### Challenge

DICOM files are large, ranging from 1 MB for a positron emission tomography scan of the heart to 1 GB for a retrospectively gated CT scan of the heart [30]. Handling imaging data is more complex compared to nonimaging data, not only due to data volume, but also due to how these data are structured, stored, and accessed. While cloud infrastructure can support scalable storage and computation, it does not by itself resolve the challenges related to efficient data retrieval and integration with other clinical data sources. Additionally, natural language processing tools such as large language models allow for the standardization and structuring of text-based information. However, this is more challenging with imaging data, as information can be burned into the pixels and hidden in the DICOM metadata.

### Mitigation

Adopting standardized data models and architectures can improve the usability of imaging data. Approaches such as linking DICOM metadata to structured databases (eg, common data models) and storing images in vendor-neutral archives enable efficient cohort identification and data retrieval. For example, extensions of the Observational Medical Outcomes Partnership common data model for medical imaging store DICOM metadata in structured database tables and link these to deidentified image files and other clinical variables [11]. This enables researchers to define cohorts using structured queries and retrieve only relevant imaging data, rather than processing entire image repositories. Scalable infrastructure, including cloud-based solutions, can support storage and computation. Standardized pipelines for data ingestion, curation, and quality control are critical to ensure consistency and reproducibility.

## Federated Learning

### Challenge

Structured EHR data is inherently suited for federated learning. However, the high-dimensional, unstructured nature of medical imaging pixels presents distinct challenges for federated learning. These are primarily centered on data heterogeneity caused by variations in scanner hardware (eg, magnetic field strengths in MRI), acquisition protocols, and institutional-specific reconstruction protocols. This statistical heterogeneity can lead to model weight divergence and hinder convergence. Furthermore, high bandwidth requirements arise from the large file size of medical imaging data. Communicating the large-scale model parameters required to process such data across a distributed network may create communication bottlenecks, particularly for resource-constrained clinical sites with limited internet speeds. Finally, the implementation of federated learning is often constrained by complex governance frameworks and a lack of standardized data management infrastructures. Many institutions lack the on-premise computational facilities or unified DICOM-compliant pipelines necessary to support decentralized model training and secure parameter exchange. These hurdles highlight that the limitations of federated learning in imaging are practical and infrastructure-related rather than a fundamental incompatibility of the data type.

### Mitigation

Recent large-scale initiatives have demonstrated that federated learning is a feasible and powerful solution for multi-institutional imaging research. For instance, the EXAM (electronic medical record chest x-ray AI model) study used a global federated approach across 20 sites to predict COVID-19 outcomes [31]. Similarly, the FeTS (Federated Tumor Segmentation) initiative has successfully applied federated learning to boundary detection in rare brain cancers, overcoming the privacy barriers that typically prevent the central aggregation of such sensitive datasets [32]. These examples suggest that the primary hurdles to federated learning in imaging are practical and infrastructure-related constraints, such as local compute capacity and standardized data curation, which can be mitigated through robust decentralized architectures.

## Deidentification

### Challenge

RWiD needs to be deidentified before it can be used for research purposes, according to the HIPAA (Health Insurance Portability and Accountability Act) in the United States and the General Data Protection Regulation in Europe [33]. For the United States, this means that the 18 HIPAA-defined sensitive data fields, such as patient name, date of birth, and social security number, need to be removed from text and images. The combination of text-based information (radiology reports), pixels (medical images), and metadata (DICOM tags) makes deidentification of this data type more complex compared to nonimaging data types. This is due to possible embedded protected health information that is not immediately apparent, such as burnt-in patient names or recognizable facial features in cross-sectional imaging [10]. Getting images out of safe and secure silos (health care providers’ IT systems) is thus more complex.

### Mitigation

Robust deidentification requires a multilayered approach addressing both metadata and pixel data. Vahdati et al [34] provide a guideline for open-source tools [35], and Willemink et al [10] describe mechanisms to prepare imaging data for machine learning purposes. Typical deidentification software uses vision and language models for optical character recognition and named-entity recognition [36]. Recently, models have been developed to identify individuals using facial recognition software, which enables the identification of individuals through facial characteristics on a 3D reconstruction of head CT or magnetic resonance [37]. Deidentification of such images can be done by deletion of facial voxels [35], facial deformation [36], face masking [38], and refacing [39]. Standardized deidentification protocols and validation procedures are essential to ensure compliance with regulatory frameworks such as HIPAA and the General Data Protection Regulation.

## Residual Confounding

### Challenge

RWiD analyses are susceptible to residual confounding, even when linked to EHR or insurance claims data. Confounding arises when differences between comparison groups influence both exposure and outcome. Confounding is common in observational studies without randomization, such as studies based on RWD [40]. Important clinical variables such as disease severity, treatment intent, or physician decision-making rationale are often incompletely captured in real-world datasets [41]. This leads to unmeasured or poorly measured confounders.

### Mitigation

While advanced analytical approaches, including machine learning and imaging-derived biomarkers, may partially mitigate this limitation, the assumption of no unmeasured confounders is ultimately unverifiable and constrains causal interpretation because residual confounding cannot be fully eliminated in observational data and should be acknowledged when interpreting results. Linking imaging data with richer clinical datasets can improve the measurement of potential confounders and reduce bias. Advanced statistical approaches, including multivariable adjustment, propensity score methods, and high-dimensional modeling, may further mitigate confounding. Sensitivity analyses should be routinely performed to assess the impact of unmeasured confounders.

## Causal Inference

### Challenge

Given these constraints, there are scenarios in which RWiD alone may be insufficient to support causal inference or regulatory-grade evidence. Observational real-world studies are particularly vulnerable to confounding by indication, where treatment decisions and outcomes are influenced by imaging usage or imaging findings [40]. More broadly, RWE lacks the randomization needed for causal inference in clinical trials, and therefore, causal relationships are inherently more difficult to establish [42]. Common causal inference pitfalls include time-zero alignment, immortal time bias, and measurement error in imaging-derived endpoints.

An incorrect definition of time zero can introduce bias when imaging, treatment, and follow-up are not aligned [43]. For example, if a study evaluating an imaging biomarker defines cohort entry at the time of imaging rather than treatment initiation and patients who deteriorate before imaging are excluded, this could inflate treatment effects.

Immortal time bias arises when patients must survive long enough to receive imaging or follow-up imaging [44]. For example, patients classified based on posttreatment CT response must survive until that scan is performed, creating an artificial survival advantage compared to those without follow-up imaging.

Imaging-derived variables are subject to variability from acquisition, reconstruction, and interpretation [26]. For example, differences in CT acquisition or reconstruction settings can alter quantitative imaging features, and interobserver variability in lesion measurements can affect response classification.

### Mitigation

Careful study design is essential to support valid inference from RWiD. Explicit definition of time zero, alignment of imaging, treatment, and follow-up, and avoidance of inappropriate conditioning on future events can reduce bias. Analytical techniques such as time-dependent modeling and landmark analysis may help address immortal time bias. Standardization and validation of imaging-derived endpoints can reduce measurement error.

## Regulatory Decision-Making

### Challenge

Consequently, for regulatory decision-making (eg, drug approval or label expansion), RWiD is generally better suited for hypothesis generation, external control arms, or postmarketing surveillance. Therefore, RWiD should be combined with other data types (eg, EHR) instead of using RWiD as a stand-alone source.

### Mitigation

To support regulatory use, RWiD should be combined with other high-quality data types, including clinical (EHR), laboratory, and outcomes data. Transparent documentation of data provenance, curation, and analysis pipelines is essential. RWiD is often most effective when used in complementary roles, such as external control arms or postmarketing surveillance, rather than as a stand-alone source of evidence.

Due to the challenges of RWiD, actual images have not been used regularly by researchers in the biopharmaceutical and life sciences sectors. Instead, researchers have typically used information captured in EHR or radiology reports without including actual images [2].

## Limitations of Radiology Reports Without Images

Imaging datasets provide direct visual evidence of disease, enabling a deeper understanding of anatomical and physiological aspects compared to textual radiology reports alone. Relying solely on textual radiology reports results in limited contextual information. Subtle changes may be missed, and interpretation of images by radiologists is known to have varying degrees of interobserver variability. Other challenges include issues such as no use of standard radiology reporting formats, poor quality or incomplete radiology reports, and inability to visually confirm findings and validate the radiology reports [45].

### Inconsistencies in Radiology Reports Due to Interobserver Variability

Interobserver variability refers to differences in the interpretation and analysis of imaging studies between radiologists. This variability leads to inconsistent radiology reports, impacting insights that can be drawn from these imaging studies. When multiple observers interpret imaging data differently, it can introduce noise into the research findings, complicating data analysis and potentially skewing results. In a clinical setting, typically multiple radiologists are working within the same institution, resulting in a variety of observers. This means that interobserver variability may decrease the validity of RWiD if only radiology reports are assessed. Addressing interobserver variability is thus crucial for ensuring robust and reproducible outcomes in clinical trials and retrospective real-world studies. As showcased by Willemink et al [46], interobserver variability can be minimized by using the actual images and training experts on a specific, standard evaluation protocol. Therefore, for accurate prospective research (clinical trials), a well-defined and standardized image analysis protocol should be used by trained experts. To improve the accuracy of retrospective research (RWE), the imaging data are needed in addition to a standardized analysis protocol.

Yoon et al [47] conducted a meta-analysis to evaluate the interobserver variability in RECIST-based tumor burden measurements and found the variability to exceed the 20% cutoff for progression. They also reported a decreased variability when RECIST was assessed by a single observer or when the sum of multiple lesions was assessed. This confirms the need for the actual images as well as a standardized analysis protocol.

### Incomplete Information in Radiology Reports

Using only radiology reports results in limited information compared to datasets that contain the actual images. Radiology reports primarily focus on clinical decision-making rather than research purposes. Radiologists evaluate images and answer the clinical question through their radiology report, which may be different from a research question. For example, Mets et al [48,49] suggest that chest CT scans for lung cancer screening can also be used for the assessment of screening for cardiovascular diseases and osteoporosis. The radiology report of a chest CT scan for lung cancer screening is typically focused on the detection of lung cancer and not on the assessment of cardiovascular diseases or osteoporosis. Additionally, radiology reports tend to miss out on detailed nuances and broader context that are useful for research. Radiology reports may lack granular details, such as shape, texture, and relationship with surrounding tissues, as radiologists focus on reporting the information that has known clinical consequences. Imaging datasets also allow for examining anatomical structures, lesions, and abnormalities in various dimensions and from multiple angles. Additionally, radiology reports typically lack information on the acquisition protocols, which tend to differ between institutions. With the actual imaging data available, researchers can ensure that only those patients will be included who underwent the desired acquisition protocols.

Disease progression definitions may vary considerably between institutions, and even within the same institution if longitudinal data over a long time span are used. For example, three major guidelines are used for pulmonary nodule follow-up, which have different recommendations [50-52]. Only using the radiology reports may thus result in skewed data, while evaluating the actual images will result in a uniform assessment. Additionally, a follow-up CT abdomen examination for a patient treated for cancer will typically contain a RECIST evaluation in the radiology report. However, additional information that is clinically less relevant, for example, atherosclerotic disease status, will not be structurally included in the radiology report. If a researcher needs this kind of information, then this can only be provided by the actual images.

### Quality of Radiology Reports

As radiology reports are produced in a clinical setting with increasing time pressure [53], the quality of these radiology reports may vary. Reduced quality can be due to factors such as time constraints, differing levels of expertise, or the focus on immediate clinical needs rather than comprehensive analysis. Radiology reports may thus lack the depth and precision required for research purposes. Imaging datasets, on the other hand, provide unfiltered data that allows researchers to apply standardized analysis methods, ensure reproducibility, and extract meaningful insights. Imaging datasets help reduce variability and enhance research rigor, enabling more precise and reliable outcomes. Garland [54] reported in 1959 a substantial error rate of 1600 of 5000 (32%) chest radiographs for the interpretation of abnormal radiographs of the chest. Subsequent studies indicated that these high error rates are still present in radiology today [55,56]. Therefore, it is essential to collect imaging data to confirm findings such as RECIST results.

## Applications of RWiD

Recent developments, such as improved imaging data handling and transfer technology, have catalyzed the ability to include the actual images in research projects [10]. Applying advanced analytics and AI methods to imaging datasets enables a variety of research and development applications [17].

## Biomarker Discovery

Biomarker discovery encompasses the identification, validation, and characterization of measurable indicators that reflect specific biological states or conditions [57]. Biomarkers may be molecular, physiological, histological, genomic, or radiographic in nature, and are widely used for diagnosis, disease monitoring, and prognostication. Medical imaging contributes uniquely to this process by providing high-resolution phenotypic information that captures structural, functional, and morphological alterations associated with disease [58,59].

Imaging-based biomarkers offer spatially resolved and anatomically contextualized insights that are not available from molecular or genetic markers alone [60]. Spatial information can reveal where pathological processes occur within tissues or organs, such as regional variations captured through parametric mapping in cardiac MRI, and thereby improve mechanistic understanding. Imaging also enables longitudinal assessment, allowing quantitative tracking of biomarker dynamics in relation to disease progression or therapeutic response.

Several clinically meaningful quantitative metrics can only be derived from imaging. Examples include coronary artery calcium scores or psoas muscle area, which, although not routinely reported, can serve as robust prognostic biomarkers. Imaging modalities have also supported the discovery of novel biomarkers across disease domains. For instance, Mastrodicasa et al [61] demonstrated how CT angiography can yield imaging-derived biomarkers in aortic dissection, showing that partial false lumen thrombosis is a predictor of mortality in patients with Stanford type B dissections [60]. Similarly, Holland et al [62] leveraged large-scale optical coherence tomography datasets to train deep learning–based clustering models for biomarker discovery in early age–related macular degeneration [56]. Their approach rediscovered established biomarkers and identified a potentially novel hypertransmission phenotype [62].

Radiogenomics uses advanced image processing and machine learning to extract quantitative features from medical images such as texture, shape, and intensity. This is correlated with underlying genomic profiles, offering a noninvasive way to capture tumor heterogeneity and biological endpoints such as genetic mutations, microsatellite instability, and hypoxia. Recent studies used radiogenomics to predict complex molecular states directly from standard-of-care imaging. For instance, PRISM-CRC (Patho-Radiomic Integrative Survival Model–Colorectal Cancer) is a notable deep-learning framework that integrates radiology (CT), histopathology, and clinical data to predict microsatellite instability status and five-year disease-free survival in colorectal cancer with high accuracy (area under the receiver operating characteristic curve of 0.91) [63].

The increasing availability of large, curated imaging datasets further accelerates biomarker discovery by enabling data-driven pattern recognition and hypothesis generation through AI. As such, imaging continues to expand its role as an indispensable source of quantitative, contextualized, and longitudinal phenotypic information for biomarker research.

## Understanding Natural History of Rare and Novel Diseases

Characterizing the natural history of a disease is essential for elucidating its progression, phenotypic variability, and untreated outcomes. Such knowledge informs mechanistic understanding, identifies critical windows for therapeutic intervention, and establishes benchmarks for clinical endpoints. In rare diseases, however, natural history studies are often constrained by limited patient populations and geographic dispersion, resulting in small, heterogeneous cohorts. Novel diseases, such as COVID-19, present additional challenges due to initial data scarcity and limited clinical experience.

Imaging plays a central role in addressing these limitations by enabling a longitudinal, quantitative assessment of structural and functional changes. Serial imaging allows investigators to monitor disease evolution with high fidelity, supporting objective measurement of progression. For example, Yu et al [64] developed deep learning models to classify progression on chest radiographs [57]. They demonstrated that automated analysis can detect interval changes and emerging pathology in pulmonary disease [64].

Integration of imaging with clinical, molecular, and histopathologic data can further uncover mechanistic insights and novel biomarkers. Vanguri et al [65] used multimodal fusion of radiographic, pathological, and genomic features to identify biomarkers predictive of immunotherapy response in nonsmall cell lung cancer [58]. Their research illustrated how imaging augments biological interpretation in heterogeneous diseases [65].

Large-scale imaging datasets also facilitate the identification of disease subtypes and variability. Jiang et al [66] leveraged brain MRI and machine learning to define four biotypes of focal temporal lobe epilepsy, each characterized by distinct neuroanatomical signatures associated with divergent long-term seizure outcomes. Functional and multiparametric imaging modalities, including ultrasound, echocardiography, and perfusion imaging, provide complementary physiological information. This enables a more holistic understanding of disease processes.

Finally, imaging-derived quantitative metrics reduce reliance on subjective assessment and improve reproducibility in natural history studies. In Duchenne muscular dystrophy, Rooney et al [67] used MRI-derived muscle biomarkers to model disease trajectory, identifying metrics capable of monitoring therapeutic effects and predicting functional outcomes. Collectively, these examples highlight imaging’s essential role in advancing natural history research. This is especially true for diseases where data scarcity or biological heterogeneity poses substantial challenges.

## Clinical Trial Design and Optimization

Effective clinical trial design requires careful selection of endpoints, patient populations, and methodological approaches to ensure validity, reliability, and statistical power. Although RWiD is typically retrospective in nature, it can inform prospective clinical trial design by providing insights into disease characterization, outcome variability, and imaging-derived biomarkers. In this context, RWiD can be considered complementary to, rather than a replacement for, randomized clinical trials.

The US Food and Drug Administration has signaled openness to novel evidence pathways that incorporate digital biomarkers, AI-derived endpoints, and nontraditional data modalities as complements to randomized trial data, including for external control arms and postmarketing commitments [6]. Imaging increasingly contributes standardized, quantitative endpoints to these frameworks. Therefore, RWiD is positioned to play a more important role in regulatory submissions.

Large imaging datasets enable the development and validation of AI models for detection, diagnosis, disease monitoring, and progression assessment. Such models can streamline trial operations through automated or semiautomated image analysis. This reduces variability and increases throughput. Imaging enables stratification of patients into biologically meaningful subgroups, supporting enrichment strategies and improving trial efficiency. For example, the MRI-derived subtypes of focal temporal lobe epilepsy identified by Jiang et al [66] could inform stratification strategies that ensure appropriate representation of biologically distinct subgroups. In oncology, Tomaszewski et al [68] demonstrated that computational analysis of preaccrual CT scans in a failed soft-tissue sarcoma trial (SARC021) could have enabled patient enrichment and potentially allowed the study to meet its primary survival endpoint.

Incorporating imaging into trial design can also support the selection of sensitive and specific biomarkers that reflect disease progression or therapeutic response. Metrics such as hippocampal volume on brain MRI in Alzheimer disease have been widely used as imaging endpoints [62]. They may also help refine inclusion and exclusion criteria to identify patients most likely to benefit from investigational therapies [69]. More broadly, imaging-derived quantitative measures, such as aortic aneurysm diameter, tumor volume, or breast tissue density, provide objective endpoints. They reduce subjective interpretation and enhance reproducibility across sites.

Collectively, these capabilities position imaging as a critical component of modern clinical trial design. They enable more precise patient selection, more informative endpoints, and more efficient trial execution.

## Participant Identification

Identifying eligible participants is a foundational step in clinical trial execution. It requires locating individuals who meet specific demographic, clinical, and phenotypic criteria. Traditional approaches, such as patient registries, partnerships with health care providers, and EHR mining, can help surface potential candidates. However, they may miss important biological or imaging-defined subgroups [70].

Imaging can be used as a screening tool to identify eligible participants at scale, particularly when eligibility depends on phenotypic features not captured in structured clinical data. As demonstrated by Jiang et al [66], MRI-based stratification uncovered four biotypes of focal temporal lobe epilepsy that share similar clinical profiles but differ markedly in neuroanatomical phenotype, highlighting how imaging can refine inclusion and exclusion criteria.

Imaging also supports more nuanced patient stratification based on predicted therapeutic response. In a retrospective analysis of multiple clinical trials in nonsmall cell lung cancer, Dercle et al [71] applied radiomics to standard-of-care CT scans to identify patients with differential sensitivity to treatments such as nivolumab, docetaxel, and gefitinib. Their findings suggest that imaging-derived signatures could enhance clinical decision-making and improve survival prediction in heterogeneous patient populations.

Beyond improving selection accuracy, imaging can accelerate large-scale screening, particularly when combined with advanced AI methods. AI models have been developed to automatically detect and characterize a wide range of conditions, including interstitial lung disease [72], vascular pathology [61], and oncological abnormalities [73]. These tools can rapidly filter imaging datasets to identify individuals meeting trial eligibility criteria, thereby reducing the burden of manual review. Imaging may be especially valuable for detecting patients with rare diseases who might otherwise be overlooked in conventional recruitment pipelines. Wojtara et al [74] describe in a review paper that recent advancements in medical imaging have enabled researchers to train AI models based on large datasets and subsequently fine‐tune these models on smaller datasets typically associated with rare diseases.

Together, these capabilities underscore the value of imaging data in expanding and refining participant identification strategies, ultimately supporting more efficient and targeted clinical trial enrollment.

## External Control Arm

An external control arm is a comparator group derived from data sources outside the randomized clinical trial, such as real-world datasets, historical cohorts, or prior observational studies [75]. This approach is particularly valuable when enrolling a traditional control group is impractical or ethically constrained, including in rare diseases and oncology. Notably, approximately 199 of 659 (30%) rare disease trials are terminated prematurely, most often due to slow patient accrual [76]. By replacing or reducing the need for prospectively enrolled placebo or control patients, external control arms can improve trial feasibility, reduce participant burden, while maintaining the ability to evaluate treatment safety and efficacy.

RWiD can significantly strengthen the external control arm construction. Imaging provides standardized, longitudinal measurements that support consistent outcome assessment and comparability between cohorts. For instance, CT imaging in idiopathic pulmonary fibrosis can detect disease progression before forced vital capacity decline or overt clinical worsening, ensuring more accurate characterization of control populations [77]. Comprehensive imaging-derived phenotypes, such as lesion size, location, morphology, and temporal trajectory, enhance the ability to match patients in the investigational and external cohorts, thereby improving comparability.

Incorporating imaging may also reduce bias. Traditional external controls often rely heavily on clinical metrics or patient-reported outcomes. This may introduce measurement inconsistencies or misclassification. Imaging-derived quantitative measures offer greater objectivity and reproducibility, strengthening internal validity and improving confidence in treatment-effect estimation. Shukla-Dave et al [78] demonstrated that RWiD increased dataset diversity and representation compared to traditional clinical trials [79]. Thereby, RWiD enabled the identification and mitigation of selection biases that cause inaccurate model performance.

Collectively, integrating RWiD into external control arm development can enhance precision, mitigate bias, and support more efficient and ethically feasible clinical trial designs.

## Postmarketing Surveillance and Evidence

Effective postmarketing surveillance relies on the integration of multiple RWD sources, including clinical records (EHR), laboratory data, and outcomes information. RWiD is most informative when linked with other data modalities rather than used in isolation. Postmarketing surveillance is essential for evaluating the safety, effectiveness, and real-world performance of drugs and medical devices following regulatory approval. By collecting data from clinicians, patients, and health systems, postmarketing surveillance efforts can detect adverse events, identify long-term outcome patterns, and inform regulatory actions such as labeling updates or the initiation of additional studies [80].

RWiD can enhance postmarketing evaluation when integrated with other RWD sources [81]. Imaging contributes detailed, objective assessments of anatomical and functional changes that complement clinical, laboratory, and outcomes data, enabling more precise monitoring of treatment effects. RWiD also offers a means to evaluate performance degradation in AI-based diagnostic devices by providing diverse, real-world image distributions over time. When linked with other data sources, such as EHR data, imaging supports a more comprehensive characterization of patient trajectories, including treatment exposure, comorbidities, and longitudinal outcomes.

Imaging further provides visual and quantitative evidence for the early detection of subtle physiological changes that may signal emerging risks or therapeutic impacts. For example, reductions in spleen volume can serve as an early predictor of radiation-induced leukopenia. Steinhelfer et al [82] demonstrated that automated splenic volume assessment in patients receiving 177Lu-DOTATATE therapy outperformed conventional laboratory parameters in predicting long-term leukopenia risk. As splenic volume is rarely measured during routine clinical care, access to raw imaging is critical for enabling such analyses.

Overall, the incorporation of RWiD into postmarketing surveillance frameworks can strengthen signal detection, refine risk assessment, and improve long-term monitoring of therapeutic and device performance.

## Drug Repurposing and Repositioning

Drug repurposing and repositioning aim to identify new therapeutic applications for existing pharmacologic agents beyond their originally approved indications. This strategy can accelerate development timelines, reduce costs, and capitalize on established safety profiles.

RWiD can meaningfully contribute to repurposing efforts by enabling direct visualization and quantification of drug effects across tissues and organ systems. Imaging allows investigators to identify and validate off-label therapeutic benefits that may not be apparent from clinical or laboratory data alone. For example, although metformin is primarily used as a first-line therapy for type 2 diabetes, imaging-based studies have shown that metformin use may slow abdominal aortic diameter expansion and reduce mortality risk following aortic aneurysm repair [83].

RWiD also enhances mechanistic understanding by capturing spatially resolved and temporal drug responses. When integrated into multimodal datasets, including EHRs, transcriptomic and proteomic profiles, and other digital health information, imaging can reveal biological pathways and phenotypic effects. These insights are relevant to new therapeutic hypotheses. Yin and Wong [84], for instance, describe multimodal pipelines for drug repositioning in Alzheimer disease and related dementias that leverage imaging alongside molecular and clinical data to identify promising candidates and understand their potential mechanisms of action.

Collectively, RWiD provides complementary phenotypic evidence that strengthens drug repurposing pipelines, supporting both discovery and validation of novel therapeutic uses.

## Conclusions

In conclusion, RWiD is an emerging and increasingly valuable asset for biopharma and life sciences. Challenges such as data harmonization, storage and transfer, computational demands, and deidentification remain. However, datasets that include actual images provide richer insight than radiology reports alone, which are optimized for clinical decision-making rather than research. When incorporated into broader RWD frameworks, RWiD enables biomarker discovery, characterization of disease natural history, more efficient clinical trial design, external control arms, participant identification, postmarketing evidence generation, and opportunities for drug repurposing and repositioning. As infrastructure and standards mature, the role of imaging in RWE generation will continue to expand.

## Abbreviations

CT: computed tomography
DICOM: digital imaging and communications in medicine
EHR: electronic health record
EXAM: electronic medical record chest x-ray AI model
FeTS: Federated Tumor Segmentation
HIPAA: Health Insurance Portability and Accountability Act
MRI: magnetic resonance imaging
PRISM-CRC: Patho-Radiomic Integrative Survival Model–Colorectal Cancer
RECIST: response evaluation criteria in solid tumors
RWD: real-world data
RWE: real-world evidence
RWiD: real-world imaging data
